# High-Throughput 1,536-Well Fluorescence Polarization Assays for α_1_-Acid Glycoprotein and Human Serum Albumin Binding

**DOI:** 10.1371/journal.pone.0045594

**Published:** 2012-09-20

**Authors:** Adam Yasgar, Silviya D. Furdas, David J. Maloney, Ajit Jadhav, Manfred Jung, Anton Simeonov

**Affiliations:** 1 NIH Chemical Genomics Center, National Center for Advancing Translational Sciences, National Institutes of Health, Bethesda, Maryland, United States of America; 2 Institute of Pharmaceutical Sciences, Albert-Ludwigs-Universität Freiburg, Freiburg, Germany; Concordia University Wisconsin, United States of America

## Abstract

Two major plasma proteins in humans are primarily responsible for drug binding, the α_1_-acid-glycoprotein (AGP) and human serum albumin (HSA). The availability of at least a semiquantitative high-throughput assay for assessment of protein binding is expected to aid in bridging the current gap between high-throughput screening and early lead discovery, where cell-based and biochemical assays are deployed routinely to test up to several million compounds rapidly, as opposed to the late-stage candidate drug profiling methods which test at most dozens of compounds at a time. Here, we describe the miniaturization of a pair of assays based on the binding- and displacement-induced changes in fluorescence polarization (FP) of fluorescent small molecule probes known to specifically target the drug-binding sites of these two proteins. A robust and reproducible assay performance was achieved in ≤4 µL assay volume in 1,536-well format. The assays were tested against a validation set of 10 known protein binders, and the results compared favorably with data obtained using protein-coated beads with high-performance liquid chromatography analysis. The miniaturized assays were taken to a high-throughput level in a screen of the LOPAC^1280^ collection of 1,280 pharmacologically active compounds. The adaptation of the AGP and HSA FP assays to a 1,536-well format should allow their use in early-stage profiling of large-size compound sets.

## Introduction

During the period from 1991 to 2000, the failure rate of drugs due to drug metabolism-related complications decreased from 40% to 11% [1], [2], [3], largely due to a concerted effort by the pharmacology field to shift the determination of absorption, distribution, metabolism, excretion, and toxicology (ADMET) properties from the development phase to the discovery and pre-clinical phases [4], [5]. During that same period, high-throughput screening (HTS) was integrated into the early stages of the drug discovery process, with the ability to identify a variety of properties of not just one compound, but hundreds of thousands of compounds in a week [6]. Early assessment of ADMET properties allows finite resources to be focused on compounds with a higher likelihood of becoming successful drugs [7], [8]. An example is *torsade de pointes*, a condition caused by QT prolongation, which has been implicated in a number of drug withdrawals [9]. To minimize this risk, teams have taken steps to integrate cardiac liability testing for compounds at the earliest stages of the discovery process [10], and recently, Titus *et al* developed a 1,536-well HTS method of profiling compounds for their potential to cause QT prolongation [11].

Considering the importance of determining drug efficacy and distribution, it is surprising that there are currently no true high-throughput assays for protein binding and there is only limited discussion of the need for such methods in the literature [12], [13]. The majority of plasma protein binding in humans can be attributed to just two proteins, human serum albumin (HSA) and α_1_-acid-glycoprotein (AGP) [3]. The importance of protein binding characteristics of drug candidates as determinants of their drug disposition has been debated [14], [15], [16]. The development of at least a semiquantitative high-throughput assay for assessment of protein binding is needed in order to bridge the gap between early- and late-stage drug discovery. Screening for larger numbers of compounds with regard to protein affinity may also serve to set up computational models for this parameter which is useful in future library design. Additionally, drugs covalently linked to albumin are very promising targeted therapeutic agents and it can be envisaged that a reversible highly potent serum protein ligand may also serve as a means to link drugs to e.g. albumin [17], [18].

**Figure 1:**
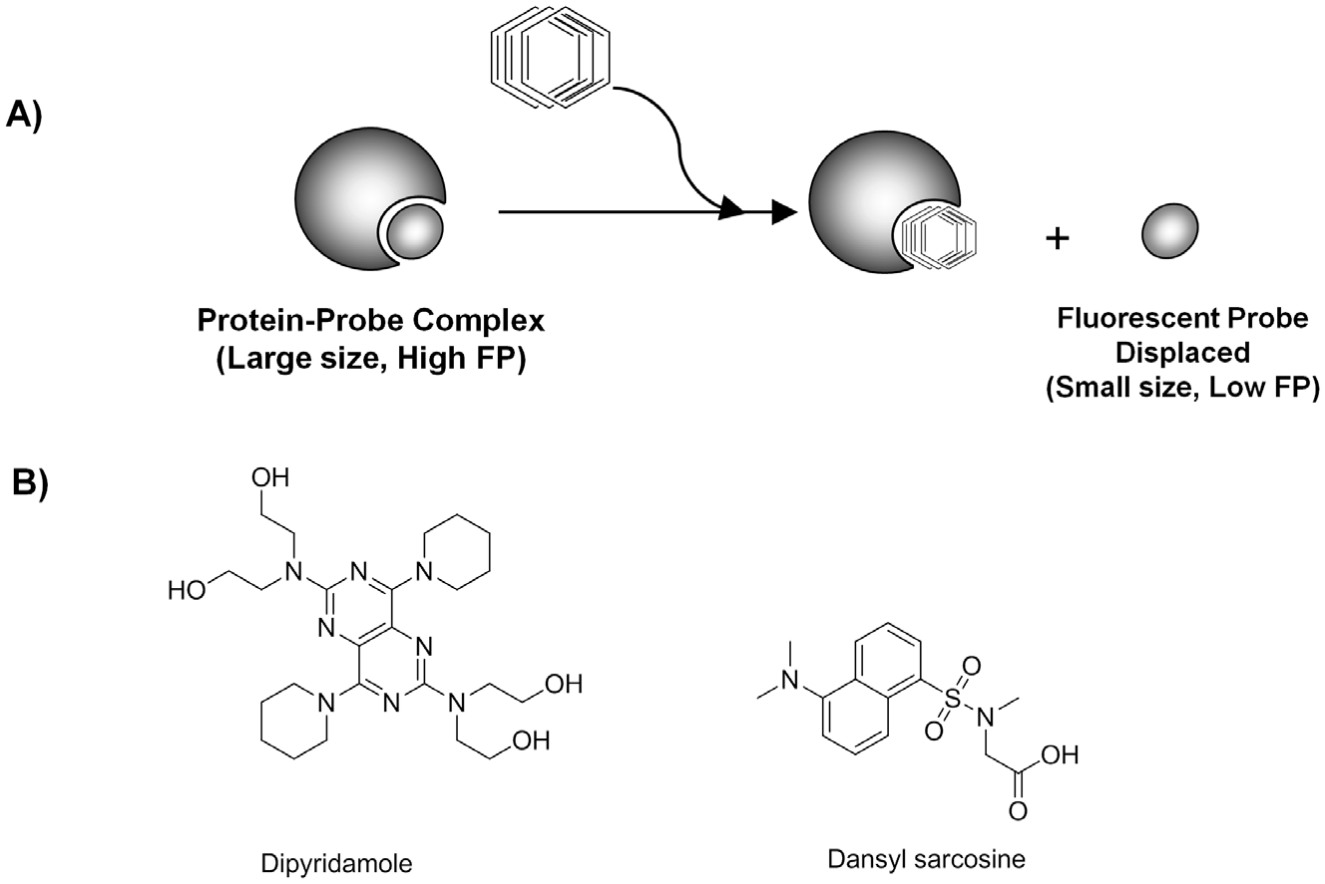
Fluorescence polarization assay. A) assay principle; B) structures of the fluorescent AGP and HSA probes Dipyridamole and Dansyl sarcosine, respectively.

Well-established methods for protein binding measurement such as equilibrium dialysis chambers, ultrafiltration, and ultracentrifugation allow for the detailed examination of one compound at a time, and thus provide reference data sets, but at the same time they make the determination of protein binding laborious and time-consuming for a large set of compounds [3], [12], [13]. In addition, most current methods require the use of radiolabeled drugs [17], [19] or low-throughput chromatographic separations [3], [19], [20], or, in the case of a recently-proposed method to predict protein binding based on enzymatic IC_50_ shifts observed upon inclusion of AGP/HSA [21], have not been validated with representative sets of compounds. In principle, a fluorescence-based method should allow for a faster throughput due to the variety of microplate readers available and their associated ability to read entire plates in seconds with a wide dynamic range. In 2007, we published the first assay format readily amenable to HTS for identifying compounds that bind to HSA and AGP [18]. The assay is based on the binding- and displacement-induced changes in fluorescence polarization (FP) of autofluorescent small molecule probes known to specifically target the drug binding sites of those two proteins. We have shown in this first study that FP is superior to using fluorescence intensity with the same probes with less susceptibility to interference by fluorescent library compounds. The initially reported exploratory study used 96-well plates and as such still suffered from low throughput; here, we describe the miniaturization of the pair of FP assays to high-density 1,536-well format (Fig. 1A). The AGP assay was optimized using dipyridamole, a fluorescent probe for the only major binding site of AGP [22]. The HSA FP assay was optimized using dansyl sarcosine, a Sudlow Site II fluorescent probe [23], [24]. The validity of the assays were determined using AGP or HSA protein-coated beads to determine binding affinity. We note that due to limitations posed by the high-throughput technology and the overall scale of the intended screens, factors known to influence protein binding [25], [26], such as the effects of temperature, pH, and ionic strength, were not investigated here.

We assembled a validation set of 10 compounds, representing binders of the Sudlow Site I or Site II of HSA [23], [24], [27], [28], and the dipyridamole site of AGP [22], respectively, and used the set to compare the miniaturized assay with the earlier 96-well method [18]. We further correlated these binding results with the corresponding K_d_ values determined using AGP or HSA protein-coated beads coupled with high-performance liquid chromatography (HPLC) analysis [29], [30]. We then utilized the pair of assays to screen a library of 1,280 pharmacologically active compounds (LOPAC^1280^) in miniaturized format.

## Results and Discussion

### Assay Principle

To date, there are no true low-volume assays to evaluate a compound’s binding affinity to the two major proteins in human serum, AGP and HSA. Here, we present the miniaturization and validation of two 1,536-well fluorescence polarization (FP) assays for AGP and HSA, respectively. The FP protein binding assay used here is based on the finding that certain intrinsically fluorescent small molecules (including several drugs) bind to AGP or HSA in a protein- or site-specific manner. Upon mixing of protein and the corresponding small molecule fluorescent probe, the fluorophore, having been engaged into a large molecular weight complex, experiences a slower rotation in solution and consequently exhibits an increased fluorescence anisotropy or polarization (FP) (Fig. 1A). In turn, a test compound which is capable of displacing the probe from the binding site will produce a low FP value due to the much faster rotation of the displaced/unbound probe [18]. Building upon our prior work in the 96-well format [18], the AGP assay was optimized using dipyridamole (Fig. 1B), a known fluorescent probe for AGP’s only major binding site [22]. The HSA FP assay was optimized using dansyl sarcosine (Fig. 1B), a known Sudlow Site II fluorescent probe [23], [24]. Site II has been recognized as the smaller and more restrictive site on HSA to which drugs, including a large category of molecules incorporating aromatic carboxylic acids with a negatively charged acidic group at one end of the molecule away from a hydrophobic center, have been shown to exhibit a higher affinity [28]. Site I was not tested because we had shown in our previous study that previously reported Site I-specific fluorescent probes did not perform adequately [18].

### Miniaturized AGP Assay

**Figure 2:**
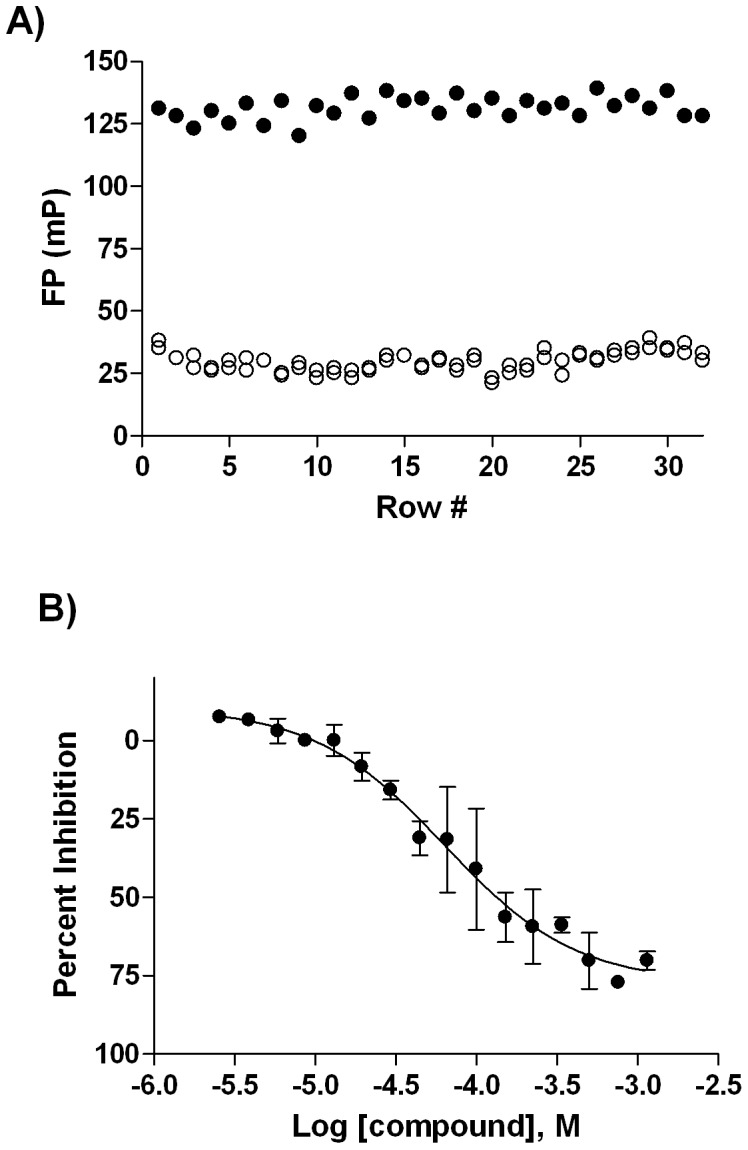
AGP Assay Miniaturization to 1536-well Format. A) whole-plate scatter plot obtained from 4 µM AGP/1 µM Dipyridamole mix (•) and 1 µM Dipyridamole (○); B) concentration-response profile of Propranolol (•).

Based on availability of optical filters and in order to better match the excitation maximum of dipyridamole, we switched the excitation wavelength of the assay from the previously utilized 340 nm to 405 nm. Titration of dipyridamole into assay buffer (to detect the minimum concentration of probe required to provide sufficient fluorescence intensity over buffer background) and of AGP against fixed dipyridamole (to establish the binding saturation) resulted in selection of 1 µM dipyridamole and 4 µM AGP as the final assay concentrations for the two binding partners (Supplemental Fig. S1). The miniaturized AGP assay was configured as described in Table 1: a Z′-factor of 0.75 and a signal window (ΔmP) of ∼101 were determined, indicating a robust performance (Fig. 2A). The known AGP binder propranolol [31], [32] exhibited dose-dependent activity in the assay (Fig. 2B) with an IC_50_ value of 63 µM, in agreement with the earlier results obtained in 96-well format [18].

**Table 1:** AGP Assay Protocol for 1,536-well Format.

Step	Parameter	Value	Description
**1**	Reagent	3 µL	AGP [4/3x] or buffer
**2**	Compound(s)	23 or 46 nL	PropranololValidation SetLOPAC^1280^
**3**	Time	15 seconds	Centrifugation at 1,000 rpm
**4**	Time	30 to 60 min	Room Temperature Incubation
**5**	Reagent	1 µL	Dipyridamole [4x]
**6**	Time	10 min	Room Temperature Incubation
**7**	Time	15 seconds	Centrifuge at 1,000 rpm
**8**	Detection	FP	Envision Read

### Miniaturized HSA Assay

**Figure 3:**
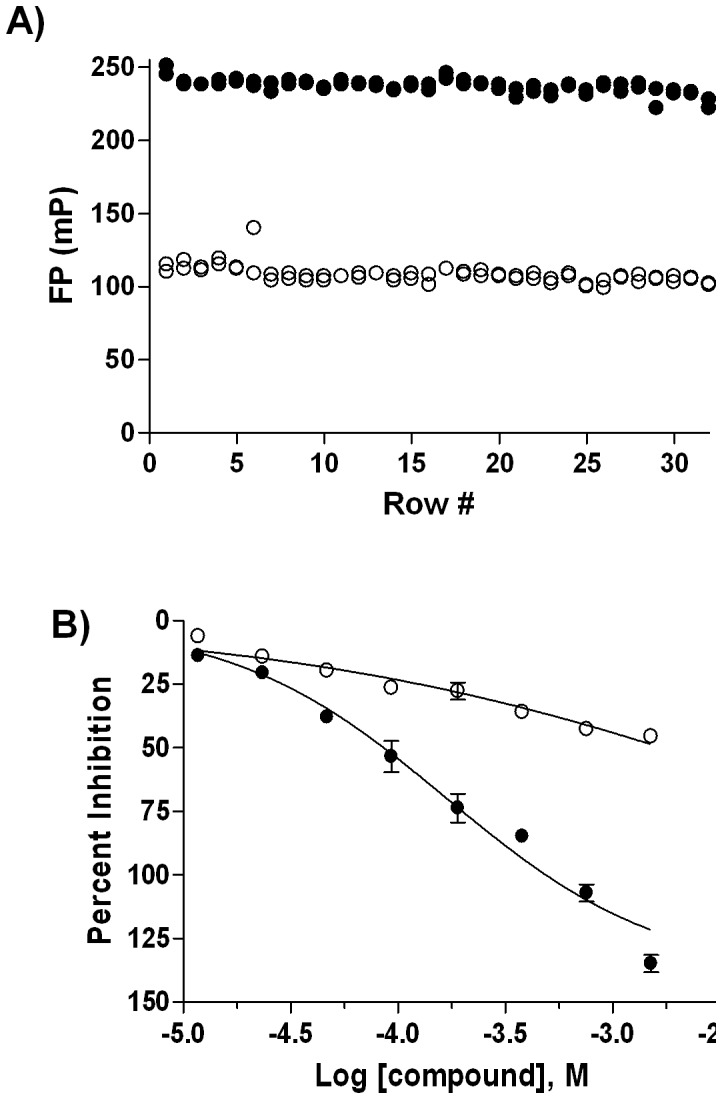
HSA Assay Miniaturization to 1536-well Format. A) whole-plate scatter plot obtained from 10 µM HSA/3 µM Dansyl sarcosine mix (•) and 3 µM Dansyl sarcosine (○); B) concentration-response profiles of Naproxen (•) and Phenylbutazone (○) in 1536-well format.

As described in our previous work [18], dansyl sarcosine was selected as an HSA probe due to its lower K_d_ and larger signal window. A matrix titration of dansyl sarcosine and HSA (Supplemental Fig. S2) resulted in the selection of 3 µM dansyl sarcosine and 10 µM HSA as the final assay concentrations, with a Z′-factor of 0.88 and an FP signal window (ΔmP) of 148 measured in 384-well plates at these conditions (Supplemental Fig. S2). The HSA assay was then miniaturized to a 1,536-well format (Table 2), producing a high Z′ value of 0.75 (Fig. 3A). The miniaturized assay was further tested using naproxen, a Sudlow Site II binder [28], and phenylbutazone, a Sudlow Site I binder [28], as positive and negative controls, respectively: the concentration-response profiles obtained here (Fig. 3B) agreed with our previously published 96-well-based studies [18]. We note that naproxen appears to be aggregating or precipitating at the highest concentrations tested here, causing the signal to decrease more than the positive control through the contribution of depolarized scattered light to the calculation of FP, which in turn leads to a calculated efficacy that is (apparently) higher than the unbound control.

**Table 2:** HSA Assay Protocol for 1,536-well Format.

Step	Parameter	Value	Description
**1**	ReagentReagent	3 µL3 µL	10 µM HSA/3 µM Dansyl sarcosine3 µM Dansyl sarcosine
**2**	Compound(s)	23 or 46 nL	NaproxenPhenylbutazoneValidation SetLOPAC^1280^
**3**	Time	15 seconds	Centrifugation at 1,000 rpm
**4**	Time	10 min	Room Temperature Incubation
**5**	Detection	FP	ViewLux Read

The physiological concentrations for both HSA (∼600 µM) and AGP (∼25 µM) are higher than those employed by us here. The main reason for this apparent mismatch in conditions is that the physiological levels are not HTS-amenable: due to the starting concentration of screening libraries (10 mM stock, leading to a maximum realistic screening concentration of 150 µM or less), at physiological protein concentrations there would be no possibility for a typical compound to displace the fluorescent probe from the protein in order to elicit assay signal. Due to the law of mass action, increasing the concentration of protein (and fluorescent probe, in order to maintain an appropriate probe fraction bound) would cause a right-shifting of the dose responses, as considerably higher compound concentration would be needed to obtain a complete displacement curve. Another reason for our inability to implement the physiological serum protein concentrations is that the requisite increase of the fluorescent probe concentration (to maintain the fraction bound required for the assay to be sensitive to displacement) would lead to a collapse of the assay due to auto-quenching of fluorescence through the inner-filter effect, as well as additional complications posed by fluorescence probe aggregation at high molarities.

### Reagent Stability

**Figure 4:**
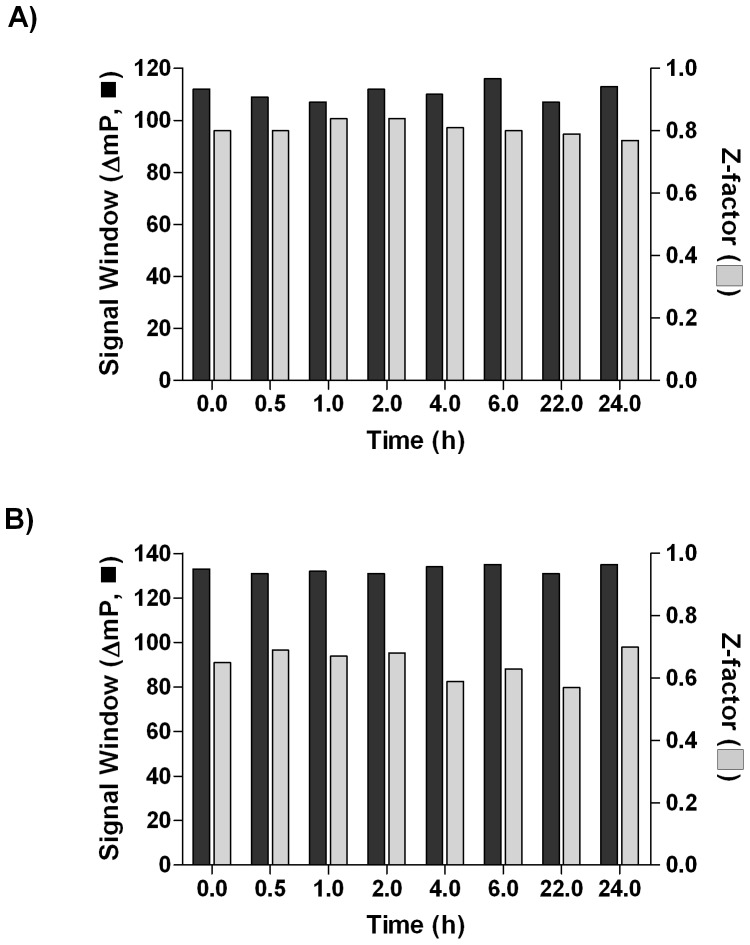
Reagent Stability as a Function of Storage Time. Signal window (ΔmP) (▪) and Z’-factor (▪) were calculated from the FP values derived from the probe-only solution vs. protein-probe mixture for AGP (A) and HSA (B).

With the final reagent concentrations chosen, the working stocks of protein and probe (i.e., AGP and dipyridamole, HSA and dansyl sarcosine) reagent components were evaluated for stability. Fresh solutions were prepared, tested at time zero, and stored at 4°C. The reagents were tested at selected time points by running the corresponding miniaturized assays. Excellent reagent integrity was observed for at least 24 hours of storage, with AGP (Fig. 4A) and HSA (Fig. 4B) assays exhibiting Z’-factor ranges of 0.77–0.84, and 0.57–0.70, respectively, indicating that an unattended overnight screen at the final reagent conditions for either AGP or HSA was feasible.

### Validation Set

**Figure 5:**
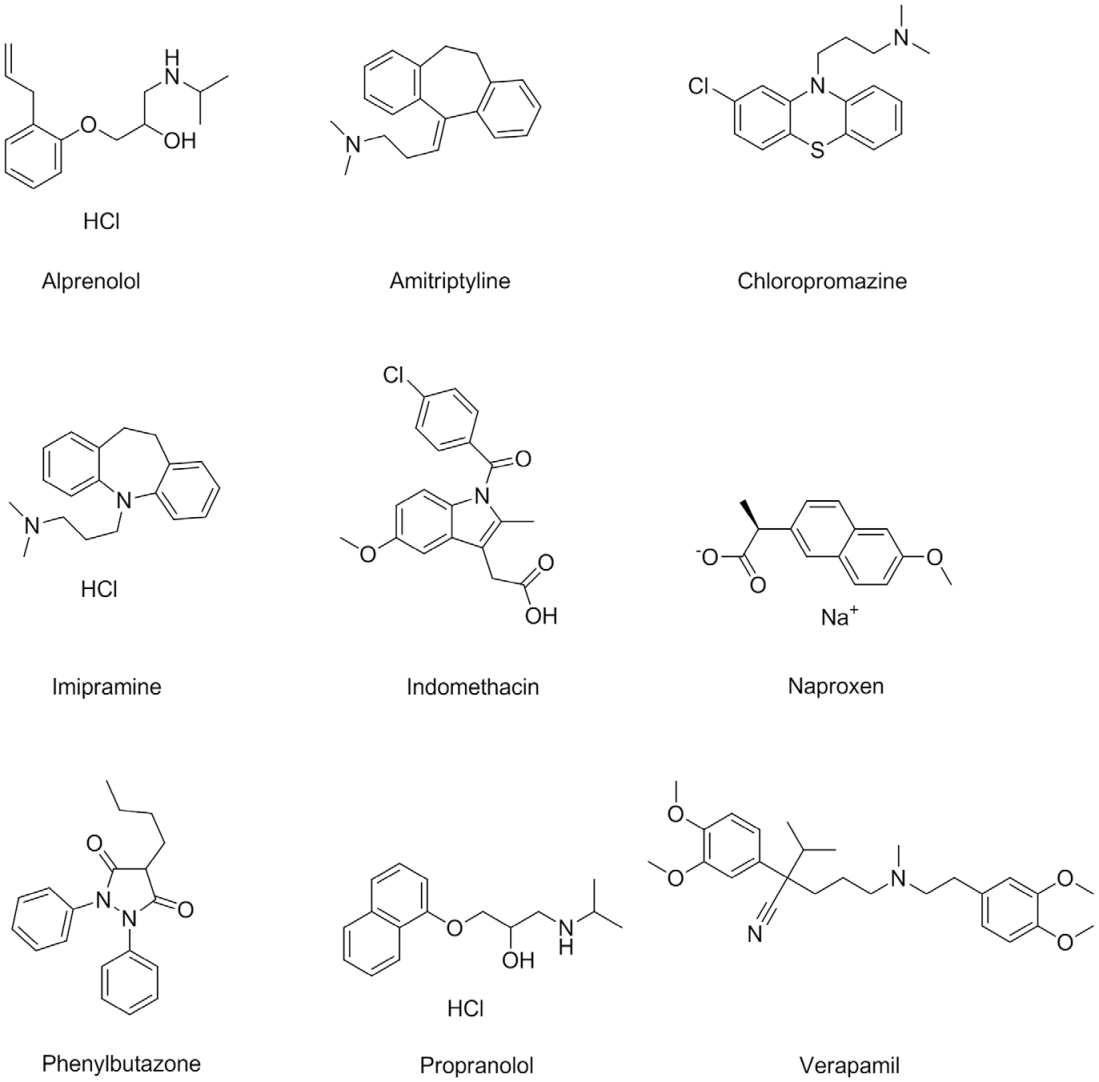
Structures of the AGP and HSA Validation Set.

Using previous reports of small molecules binding preferences to AGP and HSA [22], [33], we assembled a ten-compound validation set (Fig. 5) and used it to compare the performance of the new miniaturized assays against their 96-well-based counterparts, and against an alternative capture-and-separation based technique. The selected compounds have been determined to bind to the major site on AGP or to be HSA Sudlow Site I or II binders. The set included the AGP binders alprenolol (a β_1_- and β_2_-adrenoceptor antagonist), amitriptyline (a tricyclic antidepressant), chlorpromazine (phenothiazine antipsychotic), dipyridamole (inhibitor of platelet aggregation), imipramine (tricyclic antidepressant), propranolol (non-selective beta blocker), and verapamil (calcium-channel blocker) [18], [28], and the known HSA Sudlow Site II binder naproxen (cyclooxygenase inhibitor) [28]. Indomethacin (cyclooxygenase inhibitor) and phenylbutazone (non-steroidal anti-inflammatory) represented HSA Sudlow Site I binders [28] and were included as additional negative controls with respect to the Sudlow Site II which the present HSA assay was designed for.

**Figure 6:**
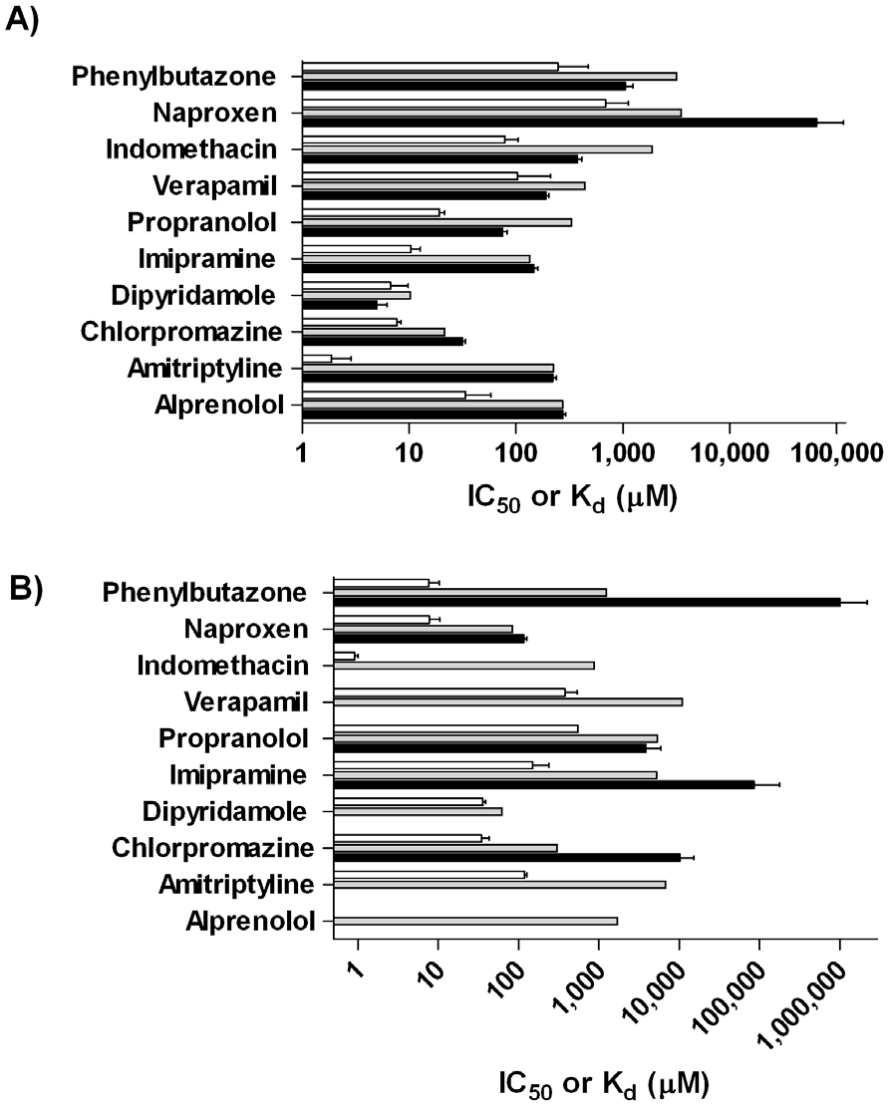
Validation Set Testing. A) IC_50_ and K_d_ values for the Validation Set in 1,536-well (▪), 96-well (▪), and protein coated bead (□) AGP assays; B) the corresponding results for the HSA assays.

In the miniaturized AGP assay, all of the reported AGP binders exhibited micromolar IC_50_ values similar to those obtained in the 96-well assay (Fig. 6A and Table 3). Having been reported [28] to bind primarily to HSA, indomethacin, naproxen, and phenylbutazone’s large, inactive, and extrapolated millimolar IC_50_ values, respectively, confirm the present assay’s ability to distinguish AGP binders from non-binders.

**Table 3:** HSA and AGP IC_50_ and K_d_ value comparisons for the 10-compound validation set.

Sample	ProteinBindingPreference^1^	AGP 96-well IC_50_ [µM]	HSA 96-well IC_50_ [µM]	AGP 1,536-wellIC_50_ [µM]	HSA 1,536-wellIC_50_ [µM]	AGP K_d_[µM]	HSA K_d_[µM]
Alprenolol	AGP	273	1,700	276	–	33.8	NC	NC	NC
Amitriptyline	AGP	224	6,751	221	–	1.89	119	63	NC
Chlorpromazine	AGP	21.5	303	31.6	10,150	7.68	34.4	4	NC
Dipyridamole	AGP	10.2	62.2	5.00	–	6.69	35.6	5	NC
Imipramine	AGP	134	5,201	146	85,480	10.4	149	14	NC
Propranolol	AGP	329	5,325	74.9	3,844	19.2	548	29	NC
Verapamil	AGP	441	10,928	190	–	29.7	410	14	NC
Indomethacin	HSA	1,879	872	376	–	78.8	0.913	NC	86
Naproxen	HSA	3,514	83.7	–	117	688	7.72	NC	89
Phenylbutazone	HSA	3,155	1,237	1,049	1,005,000	247	7.67	NC	32

–: no activity exhibited.

NC: Ratio could not be calculated.

1 Based on literature.

In the HSA 1,536-well assay, the Sudlow Site II control naproxen was active, as described earlier, while the negative controls indomethacin and phenylbutazone exhibited no activity or trace-level effect in the Sudlow Site II probe displacement assay (Fig. 6B and Table 3), as expected [18], [28]. These results demonstrate the resolution of the HSA assay between Sudlow Site I and Sudlow Site II targeting compounds. As for the known AGP binders, four of the seven tested did not show any displacement activity in the HSA assay, while the compounds that did, chloropromazine, imipramine, and propranolol, showed near-background activity, with extrapolated IC_50_ values in the millimolar range.

To demonstrate that the displacement assays reached equilibrium, the validation set was tested in different protocols where the reagents’ order of addition was varied. As seen in Supplemental Fig. S3, the IC_50_ values for the validation set compounds correlated remarkably well, with r^2^ values of 0.98 and 0.99 respectively, indicating the way in which the reagents are dispensed does not impact how the compounds interact with AGP or HSA.

### Protein-Coated Bead Assay

We verified our AGP and HSA 1,536-well assay results by determining each compound’s protein binding affinity (K_d_) to AGP and HSA protein-coated beads, with the final compound concentration in both assays at 50 µM. This system features the proteins of interest covalently bound to the surface of silica beads through the use of proprietary attachment chemistry. The surface was designed to minimize interactions with drug molecules, thus preventing non-specific binding of test substances [34]. The platform is based on a direct binding evaluation, requiring only a two-minute incubation to enable compound binding to the protein and an LC/UV or LC/MS instrument to quantitate the partitioning of compound between solution and protein-bound form; K_d_ values determined through this method have been shown to be comparable to those obtained using the equilibrium dialysis gold-standard method [26]. The K_d_ values determined for our validation set clearly define the compounds’ binding preferences for AGP or HSA, as in-range K_d_ values were returned for the cognate interactions, while the tests involving non-specific compound-protein pairs produced no detectable affinity or extremely high K_d_ values (Fig. 6 and Table 3). The K_d_ values determined by the protein-coated bead method were lower than the corresponding IC_50_ values, which is to be expected when comparing binding and displacement assays [35], [36].

The reported AGP binders exhibited AGP K_d_ values ranging from 1.89 to 33.8 µM, and HSA K_d_ values ranging from 34.4 to 548 µM (alprenolol was not determined due to its apparent inability to bind to the HSA protein coated beads). Alprenolol, amitriptyline, chloropromazine, dipyridamole, imipramine, propranolol, and verapamil exhibited higher affinity for AGP vs. HSA (Table 3), confirming their preference to bind to AGP over HSA. These data confirm the ability of the miniaturized AGP assay to identify compounds with a strong binding affinity to AGP.

The published HSA binders exhibited AGP K_d_ and HSA K_d_ values ranging from 78.8 to 688 µM and 0.913 to 7.72 µM, respectively. Indomethacin, naproxen, and phenylbutazone exhibited significantly lower K_d_ values for HSA when compared to AGP (Table 3), confirming their protein binding preference to HSA. Because the present assay reports on Sudlow Site II binders specifically, we could only interrogate that site’s binder, naproxen, which showed a distinct preference to HSA vs. AGP in the protein-coated bead assay (Table 3). Naproxen’s higher binding affinity for HSA, in combination with the millimolar activity observed by the known AGP binders in the HSA assay, confirm the capability of the HSA 1,536-well assay to determine a compound’s binding affinity to Sudlow Site II.

### LOPAC^1280^ 1,536-well Screen

After assaying the validation set, we proceeded with screens of the LOPAC^1280^ collection to assess the scalability of the assays. The LOPAC^1280^ library contains a wide array of bioactive compounds and approved drugs, and due to its accessibility is becoming a frequent starting point to identify preliminary hits for assay validation and to gauge the hit rate for future larger-scale HTS campaigns [6]. The application of the qHTS paradigm, that is the testing of every library compound at a range of concentrations, allowed us to evaluate the results based on dose response curve characteristics, such as IC_50_, goodness of fit, efficacy, and presence of asymptotes [37].

**Figure 7:**
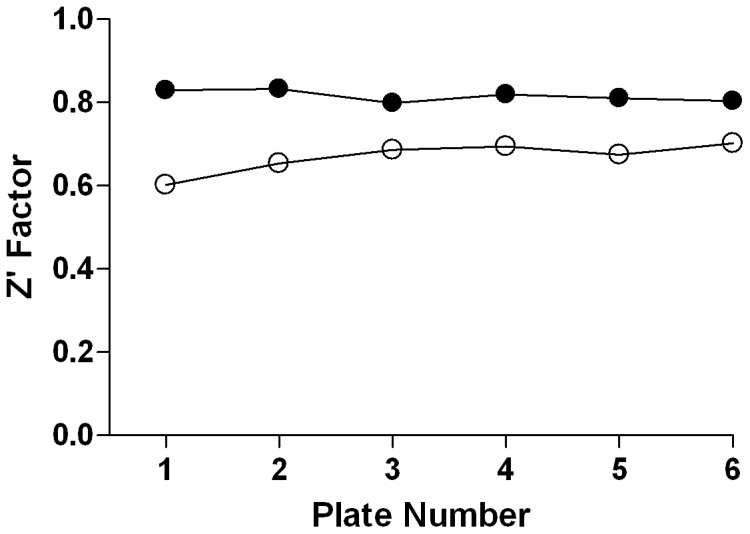
LOPAC^1280^ Screen. The robust AGP (•) and HSA (o) screen performance as represented by the reproducible high Z’-factor trend.

We used the miniaturized AGP assay to screen a six-point dilution series of the LOPAC^1280^ compound library with final compound concentrations ranging from 457 nM to 144 µM. The Z’-factor remained nearly constant throughout the experiment, with an average value of 0.81, indicating excellent assay performance (Fig. 7). The intraplate control propranolol also exhibited robust performance, yielding an average IC_50_ of 48.7 µM and a minimum significant ratio (MSR) [38] of 1.28 (assays capable of reporting a reproducible IC_50_ upon repeat testing tend to have MSR of less than 2, with MSR = 1 being a perfectly reproducible IC_50_). In turn, the miniaturized HSA assay was used to screen a six-point dilution series of the LOPAC^1280^ compound library with final compound concentrations ranging from 3.1 µM to 154 µM. The Z’-factor averaged 0.67 for the small-scale screen, again indicating a stable assay (Fig. 7). The intraplate positive control naproxen and the negative control phenylbutazone both exhibited reliable assay performance, yielding average IC_50_ values of 229 µM and >1 mM, respectively, and an MSR for naproxen of 1.77.

All 10 compounds (11 samples) used in the validation set (Table 3) were also present within the LOPAC^1280^ collection (propranolol was represented by two samples: its (*S*)-enantiomer and a racemic mixture). Of the seven known AGP binders included in the validation set, only the three highest-affinity drugs chlorpromazine, dipyridamole, and (±)-propranolol, displayed activity in the AGP LOPAC^1280^ screen, with IC_50_ values of 44.7 µM, 11.2 µM, and 50.1 µM, respectively. The three reported HSA binders (indomethacin and phenylbutazone to Sudlow Site I, naproxen to Sudlow Site II, Table 3B) [28], were also present in the LOPAC^1280^ collection and, as anticipated, were not identified as hits in the AGP assay, in a trend similar to the validation set data.

In the HSA LOPAC^1280^ screen, the only HSA validation set member to display activity was naproxen (IC_50_ 79 µM). The AGP fluorescent probe dipyridamole did exhibit an apparent IC_50_ value of 40 µM in the HSA LOPAC^1280^ screen but this was likely a fluorescent artifact, as dipyridamole’s spectrum overlaps with the range of the optics used in the HSA assay. Other reported Sudlow Site II binders present in the collection did not exhibit inhibitory response in the screen (for example, 3′-azido-3′-deoxythymidine (AZT), chlorothiazide, clofibrate, etodolac, ibuprofen, imipramine, indomethacin, and propofol [28]), most likely due to the relatively low top concentration of the library employed here.

### Testing of the LOPAC^1280^ Screening Hits in the 96-well Assay

**Figure 8:**
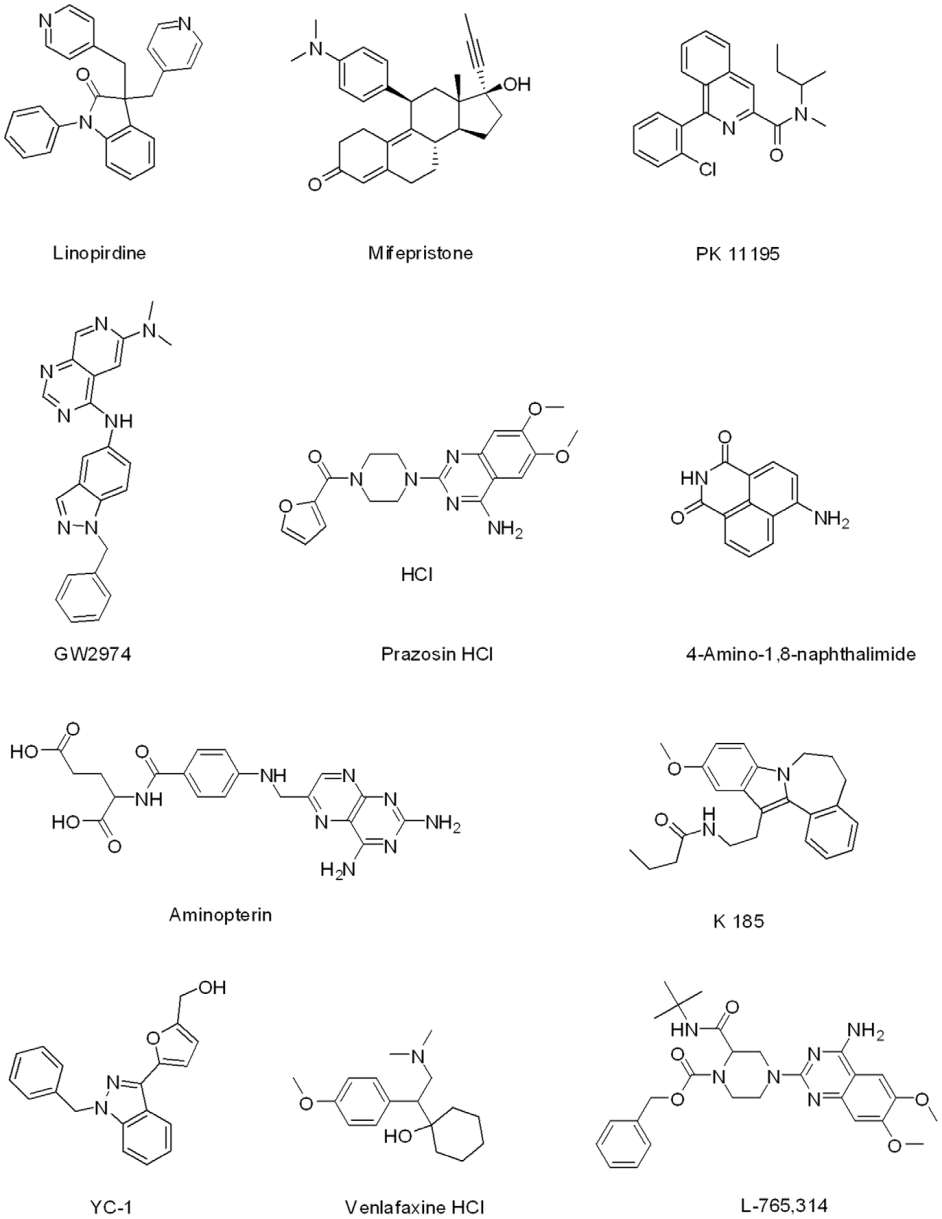
Structures of the top hits from the AGP and HSA LOPAC^1280^ Screen.

**Figure 9:**
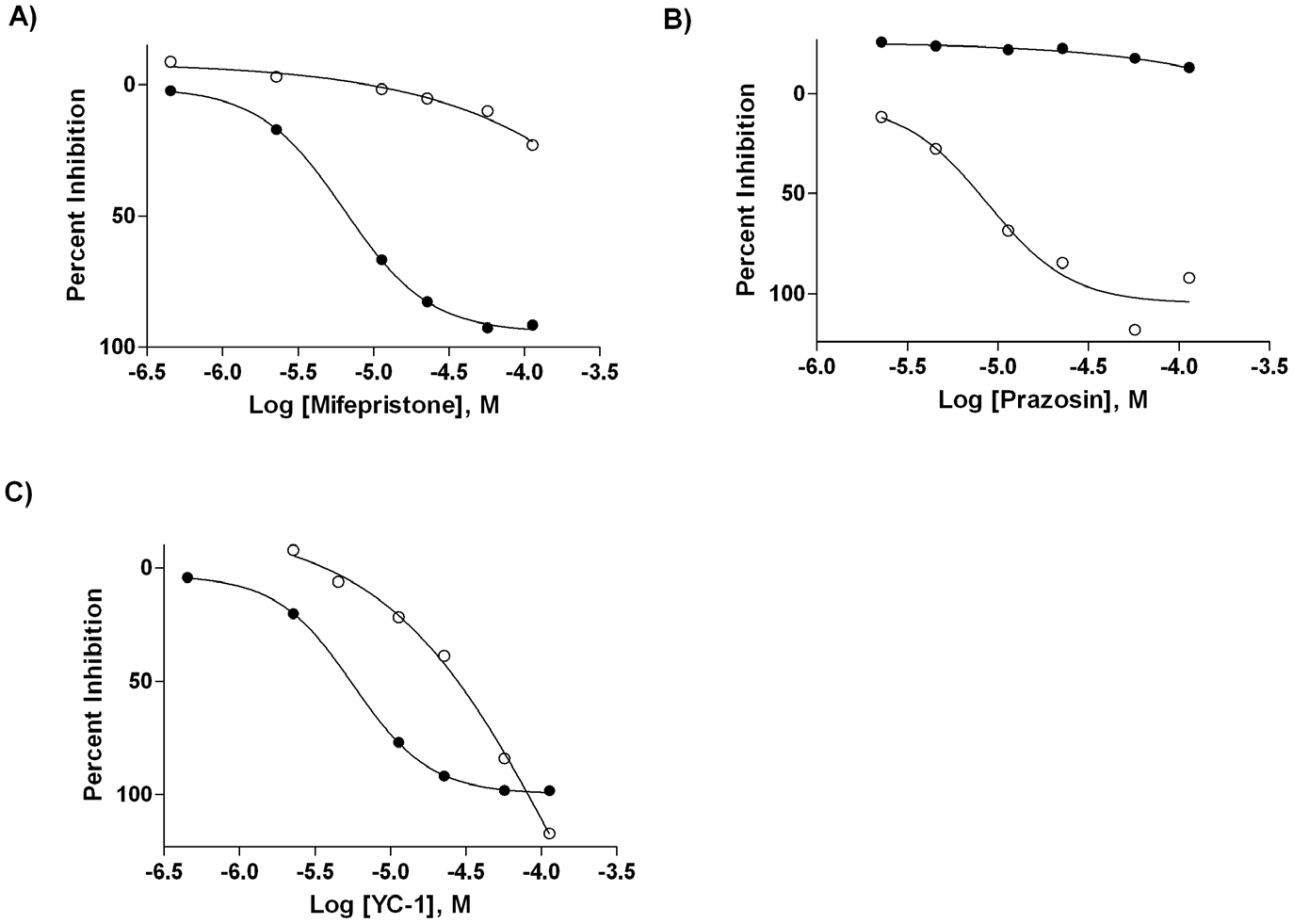
Screen-derived concentration-response curves. Concentration-response profiles of the AGP-selective hit Mifepristone (A), the HSA-selective hit Prazosin (B), and the dual-binder hit YC-1 in the AGP (•) and HSA (○) LOPAC^1280^ screens.

After analysis of the LOPAC^1280^ screening data, 11 compounds (Fig. 8, Table 4) were selected for additional testing as representatives of the categories of hits exhibiting binding preference to either only AGP (linopirdine, mifepristone, PK 11195), only HSA (GW2974, prazosin), both AGP and HSA (4-amino-1,8-naphthalimide, aminopterin, K 185, L-765,314, YC-1), or neither (venlafaxine, representing an inactive library member). Screen-derived concentration-response curves for one compound each from the AGP-, HSA-, and dual-binder categories are shown in Fig. 9.

**Table 4:** Top LOPAC^1280^ Hits.

Sample Name	Protein Binding Preference	AGP 1,536-wellScreen IC_50_ [µM]^1^	AGP 96-wellIC_50_ [µM]	HSA 1,536-wellScreen IC_50_ [µM]^1^	HSA 96-wellIC_50_ [µM]
Linopirdine	AGP	35.5	21.7	>151	ND
Mifepristone	AGP	6.31	2.51	>151	ND
PK 11195	AGP	22.4	23.9	>151	ND
GW2974	HSA	>144	ND	7.94	4.65
Prazosin	HSA	50.1	17.5	11.2	5.87
4-Amino-1,8-naphthalimide	AGP/HSA	11.2	832	7.94	37.0
Aminopterin	AGP/HSA	56.2	306	8.91	22.0
K 185	AGP/HSA	20.0	10.5	56.2	83.2
L-765,314	AGP/HSA	14.1	2.03	70.8	5.21
YC-1	AGP/HSA	5.62	4.83	44.7	5.07
Venlafaxine	None	>144	>1,000	>151	>1,000

1 Top compound concentrations of 144 or 151 µM for the AGP and HSA assays, respectively.

There was generally a good agreement between the IC_50_ values obtained from the HTS and the 96-well assays. The screening hits showing an AGP binding preference based on the HTS (linopirdine, mifepristone, PK 11195) exhibited very similar IC_50_ values in the AGP 96-well assay relative to the screen (Table 4). Similarly, for the compounds identified as higher affinity binders to HSA (GW2974, prazosin), the IC_50_ values in the HSA 96-well assay were remarkably similar to those obtained in the 1,536-well-based screen. For the hits identified in the screen as having similar affinities to AGP and HSA, 4-amino-1,8-naphthalimide and K 185 exhibited significantly right-shifted responses in the 96-well assay for AGP and were also slightly right-shifted in the HSA assay (Table 4). This trend remains unexplained at present, though most likely it is related to the autofluorescent properties of these two agents. L-765,314 and YC-1 exhibited more concordant assay results between the miniaturized screen and the 96-well testing, while for K 185 there was concordance only in the AGP assay results (Table 4). Venlafaxine reproducibly exhibited no binding in any of the assays. Efforts were unsuccessful in determining the K_d_ of the LOPAC^1280^ hits using AGP or HSA protein-coated beads described earlier as none of the compounds produced adequate signal in the LC/UV or LC/MS analytical methods utilized (data not shown).

After determining the protein binding preference of each compound based on their 96- and 1,536-well calculated IC_50_ values, a retrospective effort was made to determine how they compared to what was available in the literature. We were unable to find protein binding values or preferences in literature for 7 of the 11 screening hits, linopirdine, GW2974, 4-amino-1,8-naphtalimide, aminopterin, K 185, L-765,314, and YC-1. This shows that the assay is very suitable to identify new serum protein binders from larger screening campaigns. Mifepristone [38] and PK 11195 [39] have been shown to bind primarily to AGP, in agreement with our results. Venlafaxine also confirmed, exhibiting no binding preference [40]. Prazosin has been reported to have a higher affinity to AGP than HSA [41], [42], in contrast with our screen results, but in concordance with the retest of this hit in the 96-well assay (Table 4).

## Materials and Methods

### Chemicals and Reagents

Albumin solution from human serum (HSA, Sigma-Aldrich, St. Louis, MO), α_1_-acid glycoprotein (AGP, Sigma-Aldrich), and DMSO (Fischer Scientific, Pittsburgh, PA). Buffer was prepared from 1X PBS pH 7.4 (Invitrogen, Carlsbad, CA) and Tween 20 (Sigma-Aldrich) for a final working concentration of 1X PBS pH 7.4 with 0.01% Tween 20. The HPLC mobile phase was prepared from ammonium acetate (Sigma-Aldrich), acetonitrile (Fischer Scientific) and HPLC grade water (Fischer Scientific).

### Compounds

The following compounds were obtained from Sigma-Aldrich: (−)-naproxen sodium, (±)-propranolol hydrochloride, (±)-verapamil hydrochloride, (*S*)-propranolol hydrochloride, 4-amino-1,8-naphthalimide, alprenolol hydrochloride, aminopterin, amitriptyline hydrochloride, chlorpromazine hydrochloride, dansyl sarcosine, dipyridamole, imipramine hydrochloride, indomethacin, K 185, L-765,314, linopirdine, mifepristone, phenylbutazone, PK 11195, prazosin hydrochloride, venlafaxine hydrochloride, and YC-1.

4-Amino-1,8-naphthalimide, aminopterin, linopirdine, mifespristone, PK 11195, GW2974, prazosin, K 185, L-765,314, YC-1, alprenolol, amitripyline, chlorpromazine, dipyridamole, imipramine, indomethacin, naproxen, phenylbutazone, propranolol, venlafaxine, and verapamil were dissolved as 100 mM initial stock solutions in DMSO (except naproxen, dissolved in 50/50 DMSO/water), sonicated in a water bath for 10 minutes, and stored at −80°C.

The library of 1,280 pharmacologically active compounds (LOPAC^1280^, Sigma-Aldrich) were received as 10 mM DMSO solutions and formatted as 1,536-well compound plates of six concentrations (1∶5 dilution) at 5 µL per well. Additional details on the preparation of the compound library for quantitative high-throughput screening (1,536-well) have been previously described [37].

### Protocol for 96-well Assay

This protocol has been described in detail previously [18]. Briefly, compounds were serially diluted (16-points, 10 nM to 10 mM) and added to a mixture of dansyl sarcosine and HSA (final concentration of 500 nM and 5 µM, respectively) for a final assay volume of 250 µL. Control wells either contained a mixture of 500 nM dansyl sarcosine and 5 µM HSA (bound control) or 500 nM dansyl sarcosine (unbound control). After incubation for 30 minutes at 30 °C, samples were measured for FP at E_x_/E_m_ = 340(60)/535(40) nm using an Envision multilabel plate reader with FITC-FP mirror (Perkin Elmer, Waltham, MA) (n = 2). For AGP, compounds were serially diluted (10-points, 500 nM to 1 mM) and added to a mixture of dipyridamole and AGP (final concentration of 400 nM and 4 µM, respectively) in a final assay volume of 250 µL. Control wells either contained a mixture of 400 nM dipyridamole and 4 µM AGP (bound control) or 400 nM dipyridamole (unbound control). Samples were then measured in the same manner as described above (n = 2). For AGP or HSA, IC_50_ values were calculating using GraphPad Prism 4 (GraphPad Software Inc., La Jolla, CA).

### AGP 384 to 1,536-well Assay Optimization

We modified our protocol and evaluated the fluorescent probe dipyridamole with AGP in a 384-well format. AGP was titrated (9-points, 0 to 100 µM) with 0.4 µM dipyridamole where 20 µL of each AGP concentration (n = 3) were dispensed into a 384-well assay plate (black solid bottom, medium binding, Kalypsys/Wako, San Diego, CA), followed by a 20 µL addition of dipyridamole. The plate was centrifuged (Eppendorf 5804R, Hauppauge, NY) at 2,000 rpm for one minute and FP data were collected on an Envision at E_x_/E_m_ = 405(8)/535(40) nm (S and P channel) with FITC FP dual enhanced mirror.

For 1,536-well format (Table 1), 3 µL of AGP (bound control) or buffer (unbound control) were dispensed into a 1,536-well assay plate using a BioRAPTR Flying Reagent Dispenser (BioRAPTR, Beckman Coulter, Brea, CA). The plate was covered and incubated for 10 minutes at room temperature, followed by a 1 µL addition of dipyridamole, for a final assay volume of 4 µL. The plate was centrifuged at 1,000 rpm for 15 seconds, covered, and FP data were collected on an Envision at E_x_/E_m_ = 405(8)/535(40) nm (S and P channel) with FITC FP dual enhanced mirror.

### HSA 384 to 1,536-well Assay Optimization

An HSA titration (11-points, 0.1 to 1,000 nM) was prepared with 0.5 µM dansyl sarcosine. 20 µL of each HSA concentration (n = 3) were dispensed into a 384-well assay plate and incubated at room temperature for 15 minutes, followed by a 20 µL addition of probe. The plate was centrifuged at 2,000 rpm for 1 minute and FP data were collected on a ViewLux (Perkin Elmer) reader using a combination of UV (DUG 11 filter, E_x_ = 340(30) nm) and FITC optics (E_m_ = 540(25) nm, S and P channel) with a UV dichroic mirror. A dansyl sarcosine titration (5-points, 0 to 3 µM) was prepared in the presence of 0 µM, 10 µM, or 15 µM HSA. Samples were then processed in 384-well format as described above.

For 1,536-well format (Table 2), a 3 µL mixture of HSA and dansyl sarcosine (bound control) or 3 µL of dansyl sarcosine (unbound control) were dispensed into a 1,536-well assay plate using a BioRAPTR. The plate was centrifuged at 1,000 rpm for 15 seconds, covered and incubated for 10 minutes, and FP data were collected on a ViewLux using the optics described above.

### Compound Testing in the 1,536-well Assay

amples selected for testing were serially diluted row-wise (1∶1.5 ratio, 24 points, 8.91 µM to 100 mM, n = 2 per dilution point, 7 µL/well) [43] on a 1,536-well compound plate (polypropylene, Kalypsys/Wako). Following the above 1,536-well assay protocols for AGP (Table 1) or HSA (Table 2), a compound addition step was introduced by a pintool transfer of 23 or 46 nL from the compound plate into 3 or 4 µL of assay mixture (HSA and AGP, respectively), resulting in final compound concentrations between 135 nM–1,510 µM, and 101 nM–1,140 µM, for HSA and AGP, respectively. FP was measured 15 min after compound addition. Percent inhibition was derived from the bound (i.e., protein-probe complex), or 0% neutral control, and the unbound (i.e., free probe), or 100% displaced control, respectively, by calculating their mean values, and normalizing the data using GraphPad Prism 4.

#### Protein-Coated beads assay

AGP or HSA protein-coated beads (Sovicell, Leipzig, Germany; distributed by ADMEcell, Emeryville, CA) were thawed at room temperature for two hours. Stock solutions of each compound were diluted to a final sample concentration of 20 µM or 50 µM. Samples (45 µL) were added to 405 µL of premixed solutions of buffer and AGP or HSA coated beads and mixed thoroughly to keep the beads suspended in solution to allow for the compounds to bind to the protein coated beads [29], [30]. After centrifuging, 100 µL of supernatant were transferred to a 96-well plate and analyzed on an Agilent 1100 HPLC coupled to a diode array detector.

Aliquots of 50 µL were injected and separated using a Phenomenex (Torrance, CA) Luna 3 µm C18(2) 100A 75×3.0 mm column, maintained at a temperature of 40°C. After equilibrating the column with 50 mM ammonium acetate buffer/acetonitrile (99%/1%) at a flow rate of 500 µL/min for ∼2 minutes, compounds were detected by initiating a five minute gradient to 100% acetonitrile, held for 3 minutes, followed by a one minute gradient to 1% acetonitrile, where the composition was then maintained for the final three minutes, for a total run time of 12 minutes. The diode array detector channels were set to 230 nm, 260 nm, 300 nm, and 340 nm (bandwidth = 5 nm). Data were evaluated using the Sovicell-supplied Microsoft Excel (Redmond, WA) spreadsheets [26], [44].

### LOPAC^1280^ Screen

Both assays were screened in a similar fashion to the 1,536-well protocols described in their respective optimization sections (Tables 1 and 2) with two additional steps. After dispensing reagents, controls (46 nL; propranolol for the AGP assay, naproxen and phenylbutazone for the HSA assay) and library compounds (23 or 46 nL) were pin-transferred. For AGP and HSA, the resulting final compound library concentration ranges were 457 nM –144 µM, and 3 µM –151 µM, respectively. FP results were normalized as described above and the HTS data were analyzed as previously described, using in-house software (http://ncgc.nih.gov/pub/openhts/).

### Conclusions

We have demonstrated the miniaturization of two 1,536-well FP assays for assessment of small molecule binding to the major human serum proteins AGP and HSA. Analyses of a validation set of known binders and a pilot screen data using a diverse library of bioactives demonstrated a significant correlation between HTS-derived affinity trends and test data using lower-throughput follow-up methods. While FP, being an inherently ratiometric measurement, is able to reduce compound interference due to autofluorescence it cannot completely eliminate the problem, as observed with some of the hits here. The issue could be at least partially ameliorated through careful analysis of the individual channel (parallel- and perpendicular-polarized light) data from the FP measurement or collection of an independently-derived fluorescence data (i.e., a test where compound is added to buffer in the absence of the fluorescent probe) or, prefereably, the identification of suitable red-shifted fluorescent probes. Lastly, due to the displacement nature of the assay, we observed generally right-shifted responses leading to depressed detection limits. Nevertheless, the simplicity and scalability of the miniaturized FP assays demonstrated here paves the way to large scale profiling of small molecules for serum protein binding affinity and we have already demonstrated in this scale-up study that even among well studied drugs and chemical probes new information about serum protein binding can be identified in a high-throughput setting.

## Supporting Information

Figure S1 **Detection wavelength optimization for the AGP assay.**
(DOCX)

Figure S2 **Matrix Titration of dansyl sarcosine and HSA in 384-well format.**
(DOCX)

Figure S3 **Lack of dependence of IC_50_ values for validation set compounds on the method of assay construction.**
(DOCX)
