# Supplementary material for: High-Throughput 1,536-Well Fluorescence Polarization Assays for α1-Acid Glycoprotein and Human Serum Albumin Binding
Source: PLoS One. 2012 Sep 20;7(9):e45594. doi: 10.1371/journal.pone.0045594 (PMC3447978; doi:10.1371/journal.pone.0045594)

**Supplemental Figure S1. Detection wavelength optimization for the AGP assay.** A) Absorbance spectrum of 100 µM Dipyridamole. B) Emission spectra of 4 µM AGP and 0.4 µM Dipyridamole mixture obtained under two different excitation wavelengths. C) FP response of AGP titration against 400 nM Dipyridamole in 384-well format measured under two different excitation wavelengths.


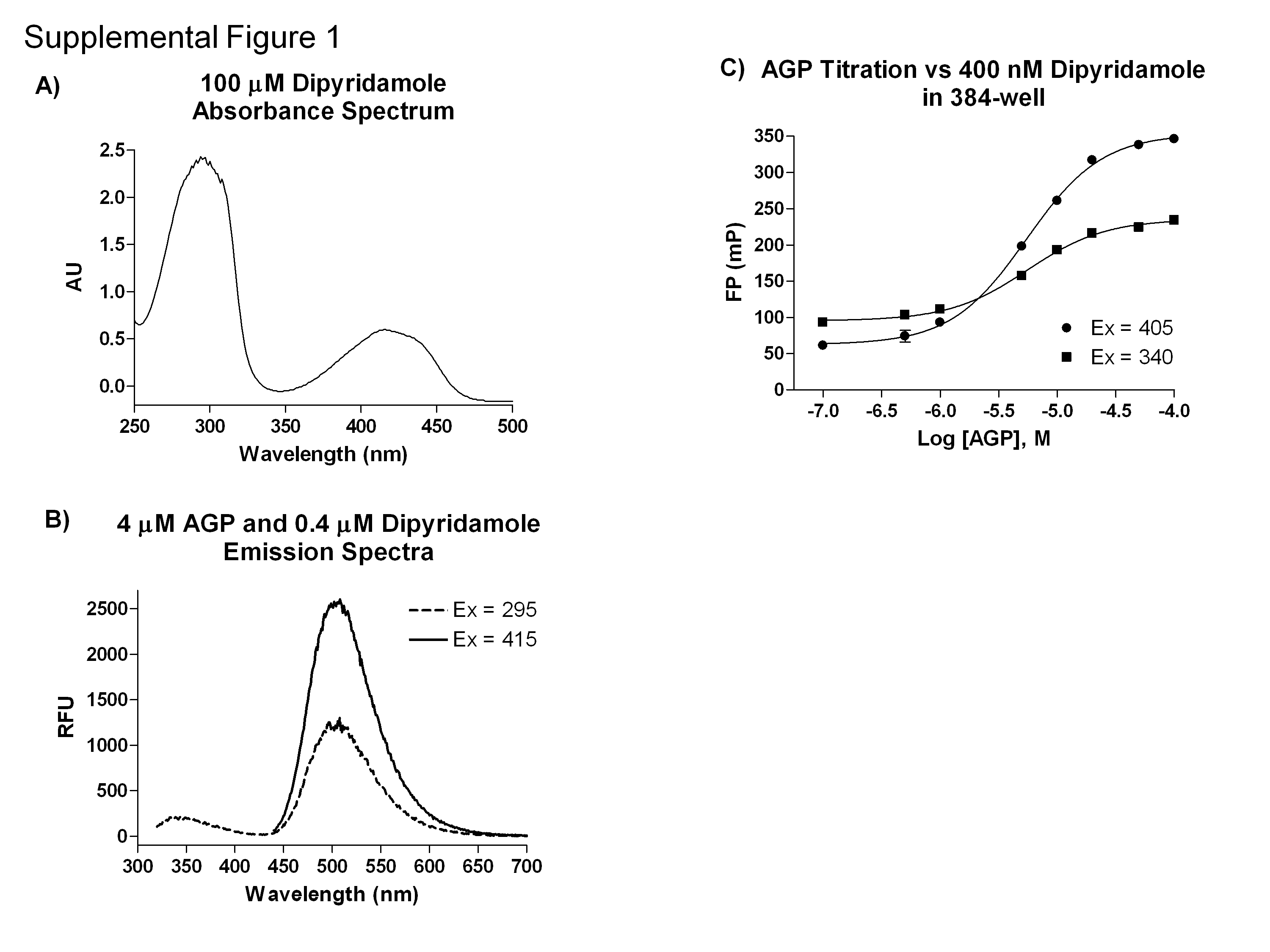


**A**

**B**

**C**


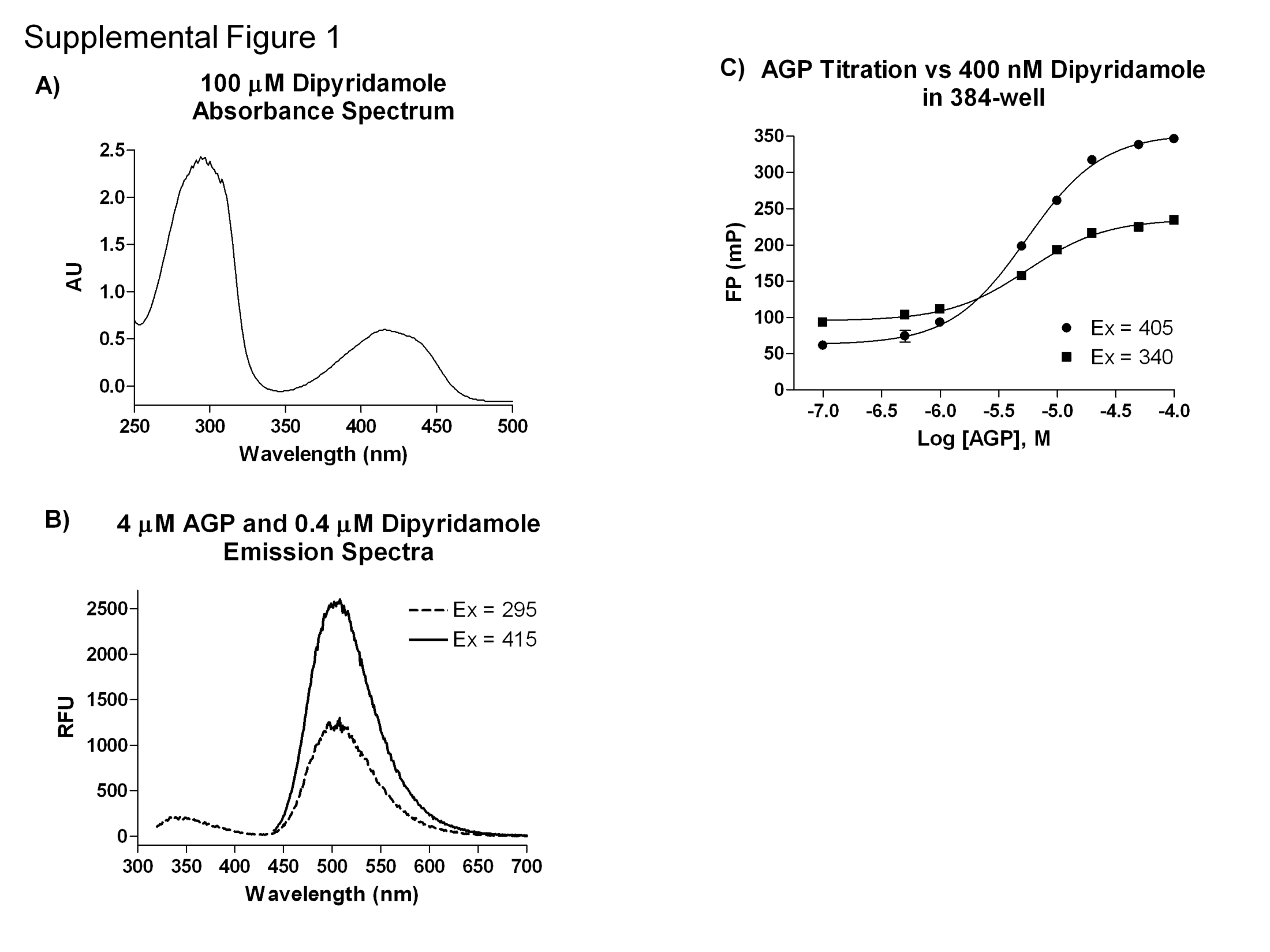

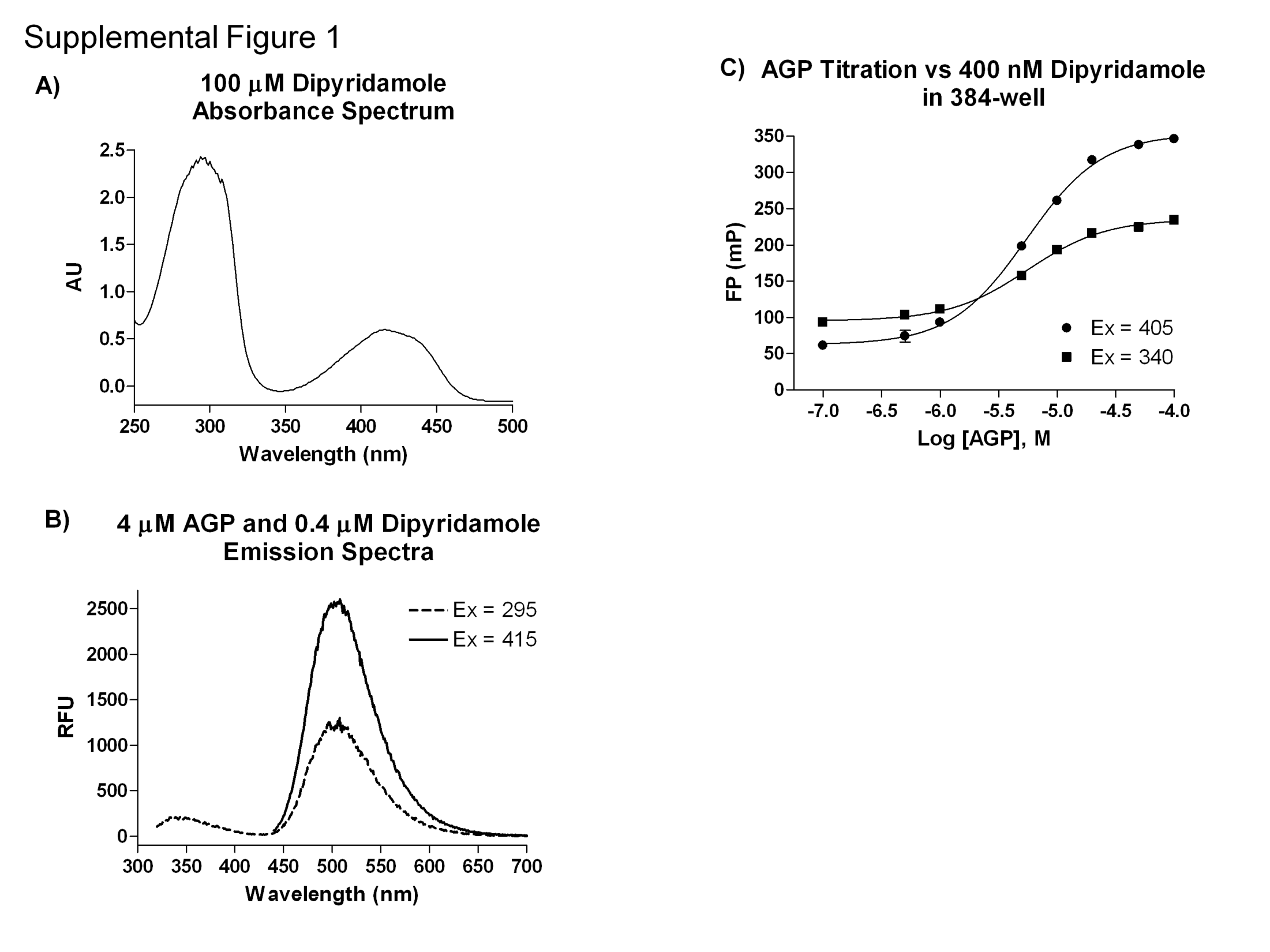

Supplement: Figure S1 — Detection wavelength optimization for the AGP assay. (DOCX) [file pone.0045594.s001.docx]
