# Supplementary figures and images for: High-Throughput 1,536-Well Fluorescence Polarization Assays for α1-Acid Glycoprotein and Human Serum Albumin Binding
Source: PLoS One. 2012 Sep 20;7(9):e45594. doi: 10.1371/journal.pone.0045594 (PMC3447978; doi:10.1371/journal.pone.0045594)

**Supplemental Figure S2.** **Matrix Titration of Dansyl sarcosine and HSA in 384-well format.**


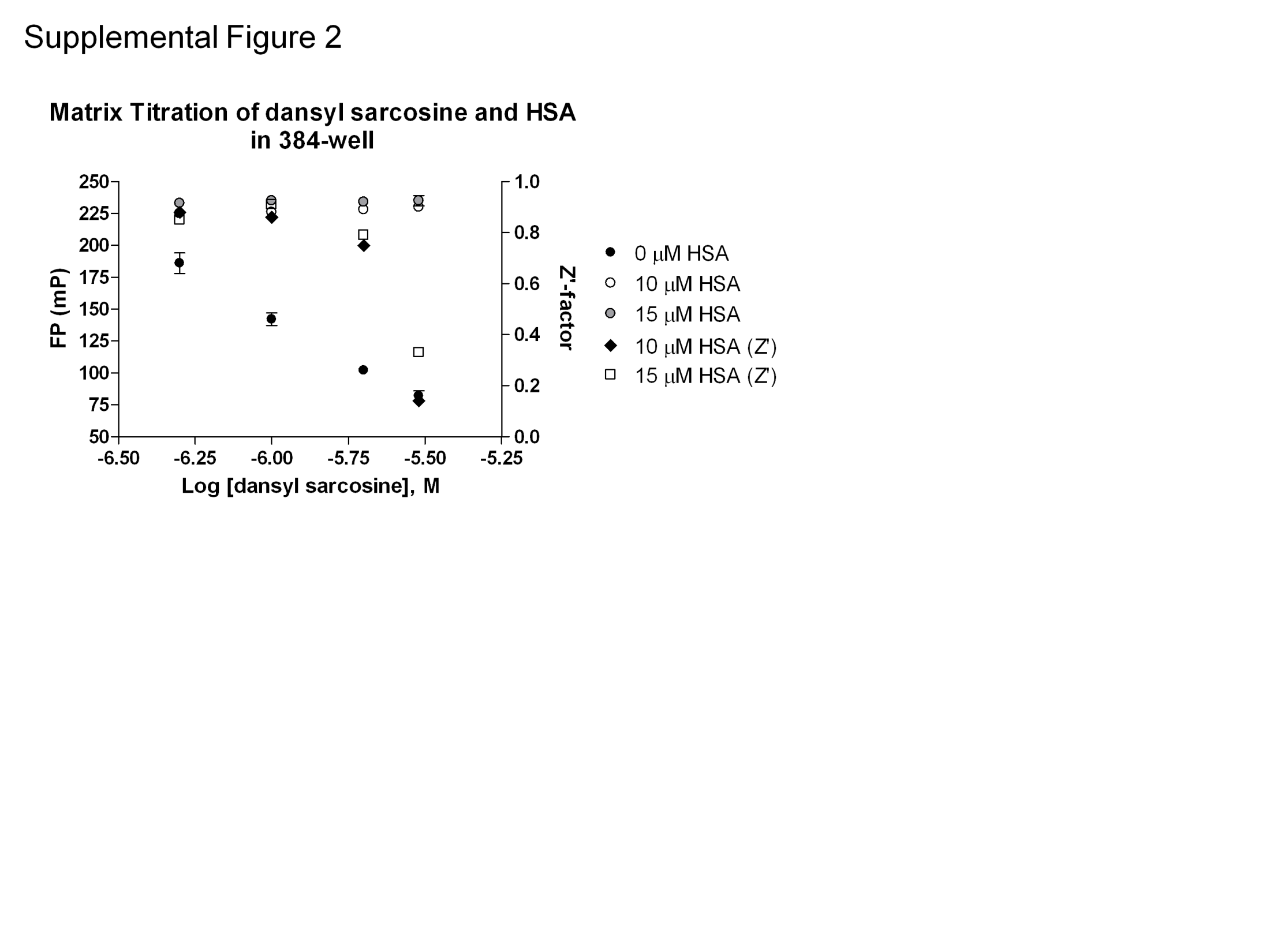

Supplement: Figure S2 — Matrix Titration of dansyl sarcosine and HSA in 384-well format. (DOCX) [file pone.0045594.s002.docx]
