# Supplementary material for: High-Throughput 1,536-Well Fluorescence Polarization Assays for α1-Acid Glycoprotein and Human Serum Albumin Binding
Source: PLoS One. 2012 Sep 20;7(9):e45594. doi: 10.1371/journal.pone.0045594 (PMC3447978; doi:10.1371/journal.pone.0045594)

**Supplemental Figure S3.** **Lack of dependence of IC_50_ values for validation set compounds on the method of assay construction.** A) AGP assay executed as a pre-mixed complex (denoted “4 µL Mix Protocol”) or as separate protein and probe delivery steps (denoted “3 µL + 1 µL Protocol”). B) HSA assay executed as a pre-mixed complex (denoted “3 µL Mix Protocol”) or as separate protein and probe delivery steps (denoted “2.5 µL + 0.5 µL Protocol”).


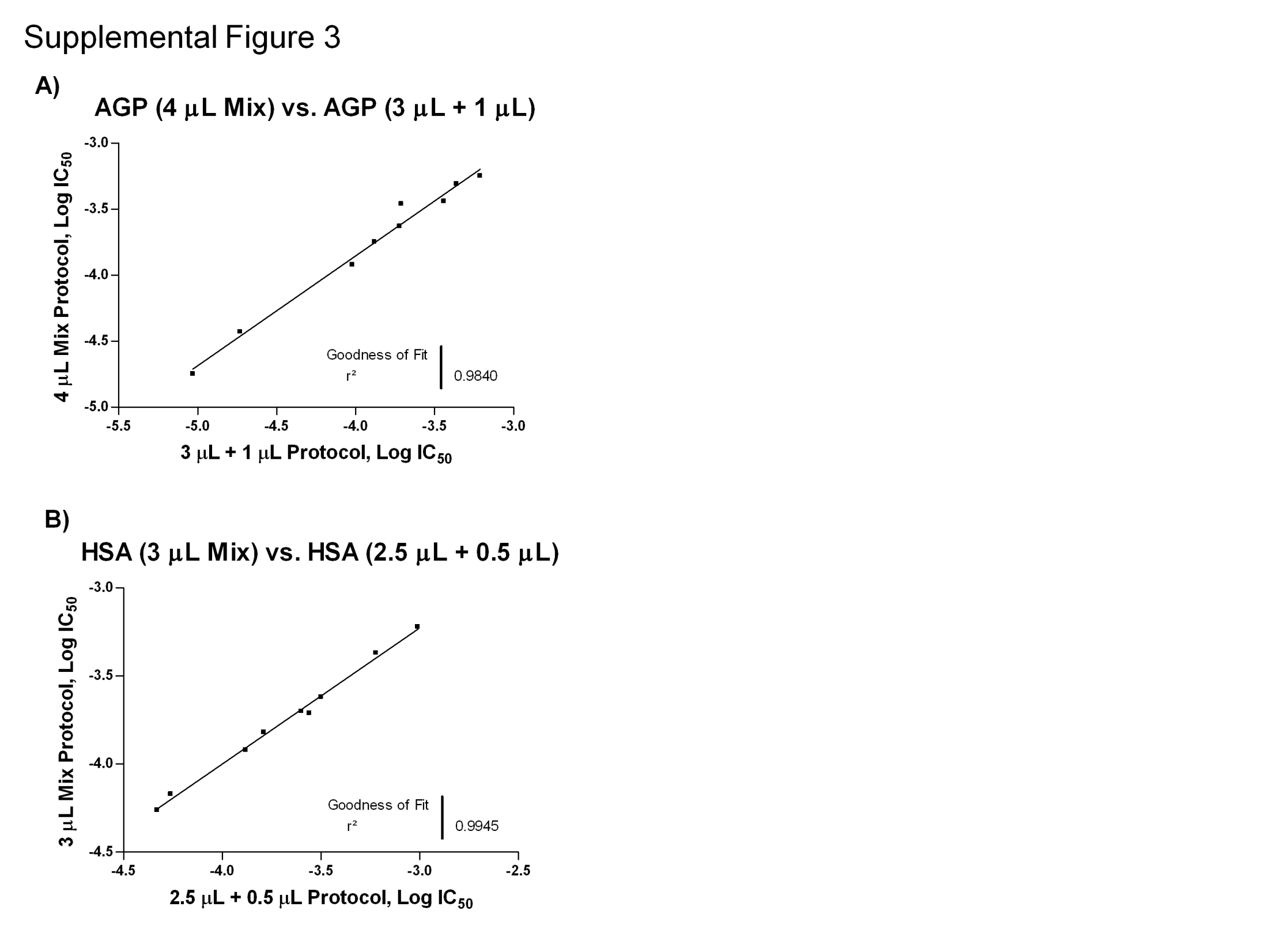


**A**

**B**


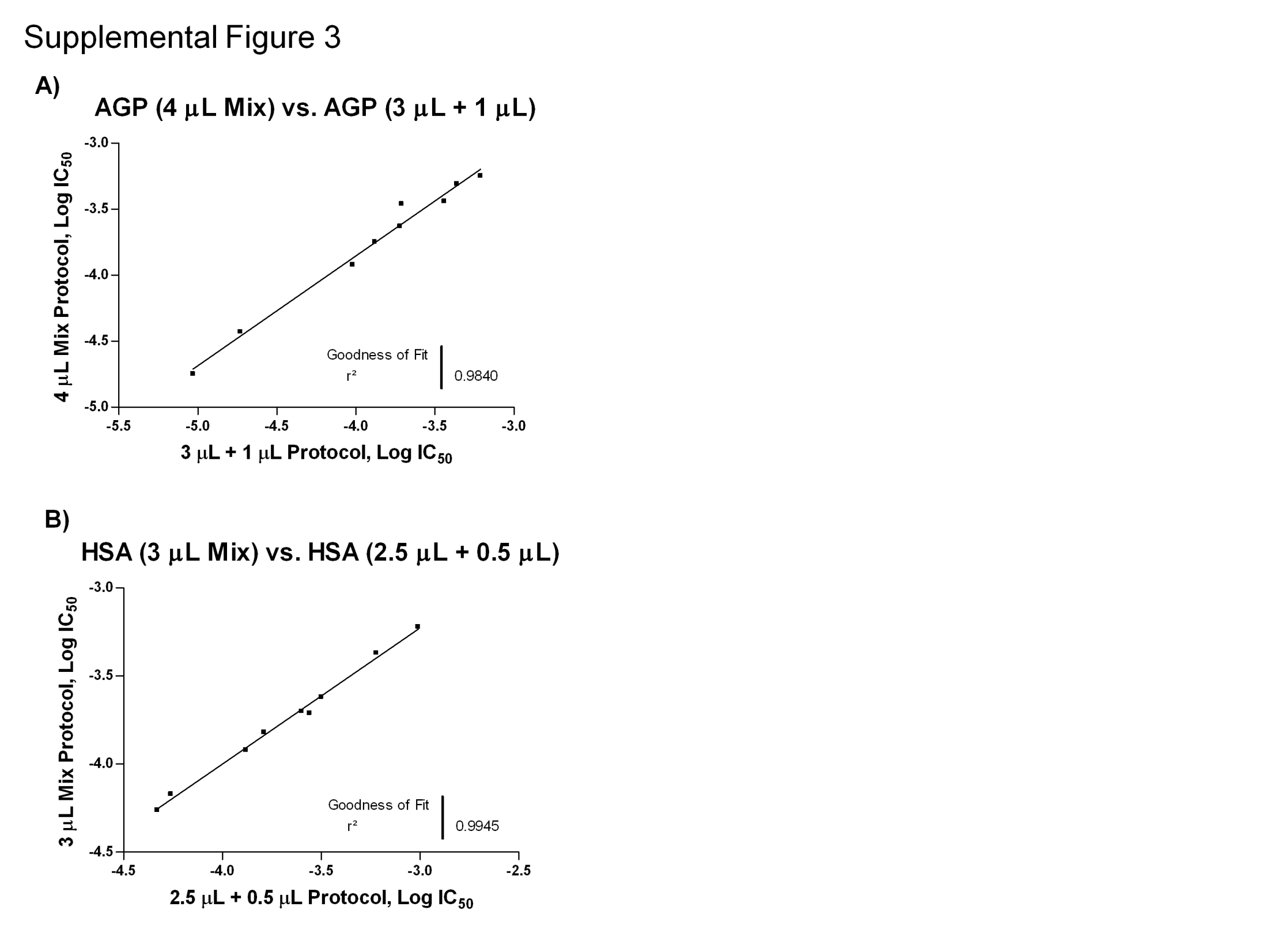

Supplement: Figure S3 — Lack of dependence of IC50 values for validation set compounds on the method of assay construction. (DOCX) [file pone.0045594.s003.docx]
